# The Crohn's-Like Lymphoid Reaction to Colorectal Cancer-Tertiary Lymphoid Structures With Immunologic and Potentially Therapeutic Relevance in Colorectal Cancer

**DOI:** 10.3389/fimmu.2019.01884

**Published:** 2019-08-22

**Authors:** Asaf Maoz, Michael Dennis, Joel K. Greenson

**Affiliations:** ^1^Boston University School of Medicine and Boston Medical Center, Boston, MA, United States; ^2^Department of Pathology, University of Michigan Medical School, Ann Arbor, MI, United States

**Keywords:** crohn's-like lymphoid reaction, colorectal cancer, adaptive immune response, tumor immunology and microenvironment, microsatellite instability (MSI), tertiary lymphoid structures (TLS), ectopic lymphoid structure, tertiary lymphoid organs

## Abstract

The Crohn's-like lymphoid reaction (CLR) to colorectal cancer (CRC), a CRC-specific ectopic lymphoid reaction, is thought to play an important role in the host response to CRC. CLR is characterized by peritumoral lymphocytic aggregates that are found at the advancing edge of the tumor. Spatial and molecular characterization of CLR within the tumor microenvironment (TME) have uncovered a spectrum of peritumoral lymphoid aggregates with varying levels of organization and maturation. In early stages of CLR development, CD4+ T-cells cluster predominantly with mature antigen presenting dendritic cells. As CLR matures, increasing numbers of B-cells, as well as follicular dendritic cells are recruited to create lymphoid follicles. When highly organized, CLR resembles functional tertiary lymphoid structures (TLS), allowing for lymphocyte recruitment to the TME and promoting a tumor-specific adaptive immune response. CLR has been consistently associated with favorable prognostic factors and improved survival among CRC patients, often providing more prognostic information than current clinical staging systems. However, consensus is lacking regarding CLR scoring and it is not clinically assessed or reported. Differences between CLR and other cancer-associated lymphoid structures exist both in primary and metastatic disease, underscoring the need to characterize organ-specific TLS. Further research is needed to explore the role of CLR in predicting response to immunotherapy and to leverage CLR to promote immunotherapeutic strategies in CRC.

## Introduction

The immune response to cancer, and specifically colorectal cancer (CRC), has profound molecular, biological and clinical implications. Immune cell populations in the tumor microenvironment (TME) are associated with distinct molecular events (e.g., mismatch repair deficiency), histopathological features, and overall and cancer-specific survival (1–6). In colorectal cancer, the adaptive immune response, in the form of lymphocytic infiltration of the tumor epithelium, the peri-glandular tumor stroma, and the peritumoral microenvironment, have all been associated with clinical outcomes (3, 4, 7). The Crohn's-like lymphoid reaction (CLR) to CRC is characterized by peritumoral lymphocytic aggregates that are found at the advancing edge of the tumor. CLR, identified over 25 years ago (8), bears structural and functional similarities to other cancer-associated tertiary lymphoid structures but has distinct features and clinical implications.

This review will present the pathological, molecular and clinical features associated with CLR. It will also cover the role of CLR in the adaptive immune response to cancer, mechanistic insights from murine models and a comparison with other cancer-associated tertiary lymphoid structures.

### Histopathological Assessment of the Crohn's-Like Lymphoid Reaction to Colorectal Cancer and Its Relation to Survival

**Figure 1:**
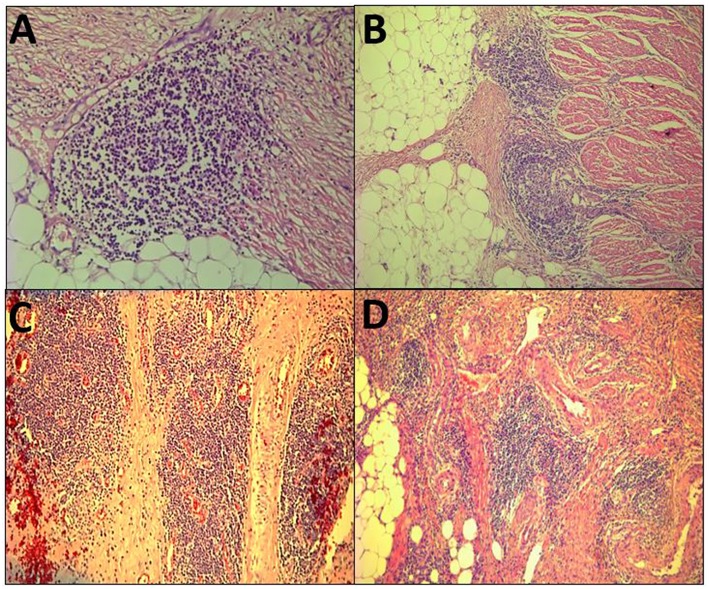
Histopathological view of the Crohn's-like lymphoid Reaction. **(A–D)** demonstrate the variable pathologic appearance of the Crohn's-like lymphoid reaction.

The term Crohn's-like lymphoid reaction (CLR) was coined by Graham and Appelman at the University of Michigan in 1990 (8). CLR was described as discrete lymphoid aggregates, some with germinal centers (GC), mostly located in the muscularis propria and pericolonic adipose tissue beyond the advancing tumor edge (Figure 1). When initially described, the intensity of CLR was associated with survival and it was hypothesized to be a favorable host response to CRC. Similar structures have been associated with non-CRC cancers and have been referred to as ectopic or tertiary lymphoid structures (ELS or TLS) (9). However, the criteria that have been proposed to define TLS, such as having distinct B- and T-cell zones with evidence of GC activity (9), had not been applied to many of the studies of CLR. Hence, this review will use the term CLR, recognizing that there is no known biological relationship between CLR and Crohn's disease, and that CLR could be regarded as CRC-specific ELS/TLS. CLR should be distinguished from tumor draining lymph nodes (TDLN), which are secondary lymphoid organs and represent a different compartment of the immune response to cancer.

Different methods for quantifying the intensity of CLR have demonstrated prognostic relevance, including implementing the semi quantitive approach (3, 10) originally suggested by Graham and Appelman, as well as dichotomous scales based on the diameter of the largest lymphoid aggregate or the density of CLR in the invasive front of the tumor (11–13). An immune score for CLR has also been proposed based on CLR density and maturation (14). Notably, this differs from the Immunoscore project that has been validated by Galon et al. which measures CD3+ and CD8+ T-cell density but does not incorporate the structural organization of these cells. The Immunoscore by Galon et al holds promise for integrating the host immune response into CRC clinical staging criteria (15). Unfortunately, there is currently no consensus on the preferred method for measuring and reporting CLR and it is not routinely incorporated into clinical practice.

Multiple studies have validated the link between CLR and CRC outcomes (7, 10, 11, 16–19). CLR has been associated with lower incidence of locoregional recurrence, fewer distant metastases and better cancer-specific and overall survival (3, 13, 14, 20). There is data to suggest that CLR scoring provides as much or more prognostic information than other biomarkers. In multivariate statistical models predicting survival, CLR had a greater impact on survival prediction than conventional histologic parameters such as tumor differentiation and venous invasion (13); tumor infiltrating lymphocytes (TILs) (7); or microsatellite instability (3). Remarkably, TILs and CLR provided more prognostic information than TNM classification in CRC that has not spread to lymph nodes or beyond (3). These data suggest that CLR is a highly valuable prognostic marker that should be further explored in prospective trials.

### Clinical and Pathological Correlates of the Crohn's-Like Lymphoid Reaction

In addition to the prognostic significance of CLR, it is associated with important molecular and immunological characteristics. Tumors that are deficient in the DNA mismatch repair system (dMMR), leading to microsatellite instability (MSI-H) and a high mutational burden, are associated with the presence and density of CLR (1, 2, 12, 21). This association is clinically important because identification of MSI-H tumors is an important step in detecting patients who are at risk for Lynch Syndrome, the most common form of hereditary colorectal cancer. Additionally, eligibility for immune checkpoint inhibitors (ICI) in metastatic CRC is determined based on MSI or MMR status. However, only 30–50% of MSI-H/dMMR patients respond to ICI (22) and further markers to predict response to ICI are needed. The relationship between CLR and the consensus molecular subtypes of CRC, or other hypermutated phenotypes such as Polymerase Epsilon-associated CRC, has not been well-characterized. SMAD4 alterations have been associated with the absence of CLR (23). A tumor suppressor, SMAD4 is often downregulated in the Canonical molecular subtype of CRC (CMS2), which lacks strong immune activation (24, 25).

In regards to immunological features, CLR has been consistently associated with increased tumor intraepithelial and stromal lymphocytic infiltrates (3, 12, 26), an association that appears to be independent of MSI status (3). CLR density is also correlated with peripheral immune markers, such as the absolute peripheral lymphocyte count and the lymphocyte to neutrophil ratio (14), which themselves carry prognostic significance (27). Recently, CLR has been shown to be the only lymphocytic component that is inversely correlated with PD-L2 expression (28). In addition, CLR promoting chemokines are inversely associated with intratumoral expression of immune checkpoint molecules, including PD-1, PD-L1, and CTLA-4 (29).

CLR is predictive of response to chemotherapy in the context of metastatic CRC, suggesting a potential synergistic effect of cytotoxic chemotherapy with the adaptive immune response to metastatic disease (30, 31). In the era of cancer immunotherapy, the role of CLR as a histological marker that could predict response to ICI or other immunomodulatory agents merits investigation.

### Initiation and Maturation of the Crohn's-Like Lymphoid Reaction

**Figure 2:**
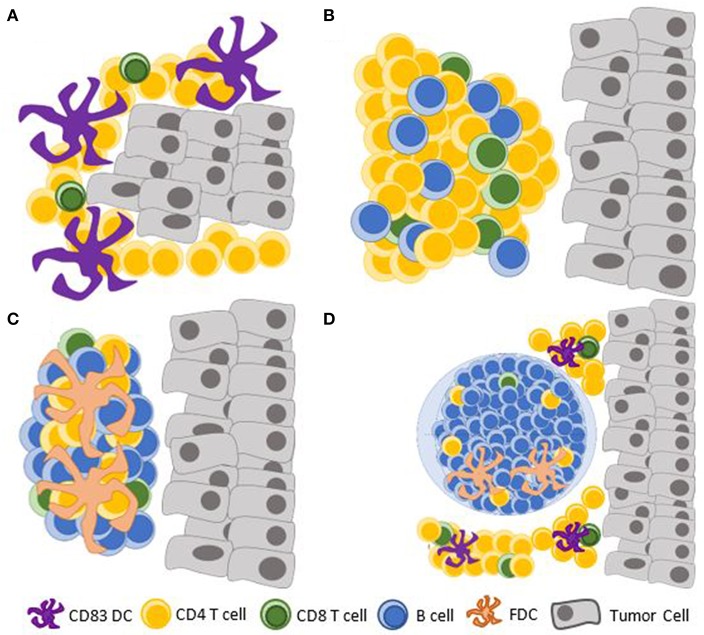
Documented maturation stages of the CLR. **(A)** Clusters of mature CD83+ DCs with predominantly CD4+ T-cells; **(B)** Dense lymphocytic aggregates form without FDCs; **(C)** Primary follicle-like TLS, with an increasing abundance of B cells and FDCs, but no germinal center reaction; **(D)** Secondary follicle-like TLS, having active germinal centers. TLS, tertiary lymphoid structure; DC, dendritic cell; FDC, follicular dendritic cell.

There exists a spectrum of CRC-associated lymphoid aggregates ranging from unorganized lymphocytic clusters to highly organized TLS containing GCs (Figure 2). A single peritumoral lymphoid follicle can be found in as many of 95% of CRC and is often associated with lymphatic vessels (12, 14, 32). Small lymphoid aggregates of <30 lymphocytes, including some that are not detectable on standard hematoxylin/eosin staining, have been shown to contain predominantly CD4+CD45RO+ T-cells with associated antigen presenting CD83+ mature dendritic cells (DC) (33). These may represent the earliest stage of CLR formation that has been documented. Immature CD1a+ or CD11c+ DCs can be found at this early stage within the tumor and the invasive margin, but they do not preferentially form clusters with lymphocytes (33–35). These data suggest that early events in the formation of CLR include tumor antigen uptake by immature DCs, which undergo maturation and form clusters with CD4+ T-cells. This sequence is supported by murine models of viral-induced lung ELS formation, in which lymphocyte-DC aggregates preceded the formation of organized lymphoid structures (36, 37). In this model, CD11c+ DCs, through secretion of lymphotoxin beta and additional chemokines known to promote CLR (38), were also needed for the maintenance ELS (39). A mouse model of CLR, induced by modified DCs that were injected into a subcutaneously-planted CRC cell line, also showed that T-cell infiltration is an early event in CLR formation, preceding recruitment of B-cells to the TME (40). Data are lacking regarding the role of Lymphoid tissue inducer (LTi) cells in CLR; LTi cells have been implicated in lymphoid organogenesis in other models (41, 42).

Several chemokines drive the formation and maintenance of TLS across different cancers, including CXCL13, a selective B-cell attractant (43) that plays an important role in attracting LTi cells; as well as CCL19 and CCL21, involved in LTi attraction, and lymphocyte and dendritic cell homing (42). These chemokines have been validated in CRC and with other chemokines, establish a TME transcriptional signature that is associated with CLR (38). The other chemokines comprising this gene signature include CXCL9, CXCL10, and CXCL11, T-cell attractants; and CCL2, CCL3, CCL4, CCL5, CCL8, and CCL18, chemoattractants for monocytes/dendritic cells, T cells, B cells, and NK cells (38). IL-36γ has been shown to be important for formation and maturation of TLS in a murine model of DC-induced CLR; in human CRC, IL-36 is secreted predominantly by M1 macrophages and the vasculature, and is associated with a CD4+ central memory T-cell infiltrate and an increased density of B cells in CLR (29, 40).

Maturation of CLR, as of other TLS (44), has been evaluated based on the structural similarities it bears to secondary lymphoid structures (45). It has been suggested that CLR has three maturation stages with functional and prognostic implications: [1] Early TLS, composed of dense lymphocytic aggregates without follicular dendritic cells (FDCs), [2] Primary follicle-like TLS, having FDCs but no GC reaction, and [3] Secondary follicle-like TLS, having active GCs (14). These histologic findings may be altered in the context of neoadjuvant chemotherapy or steroid treatment, as demonstrated in squamous cell lung cancer (44). While this has not been shown in CLR (46), neoadjuvant chemotherapy is indicated in certain clinical scenarios of locally advanced rectal cancer and may affect the study of CLR in rectal cancer.

### Structure and Function of Mature Crohn's-Like Lymphoid Reaction

The hypotheses and experimental data that support similar functions of TLS, such as CLR, and secondary lymphoid structures, have been reviewed in detail (9, 45, 47). Prominent CLR, which is defined differently by different authors, can be found in over 30% of CRC cases (32, 48). Larger lymphocytic aggregates have varying numbers of CD8+ T-cells as well as CD20+ B-cells (33), which preferentially proliferate within GCs (12). Averaged over 160 CLR aggregates from five patients, Vayrynen et al. found that the largest subset of cells comprising CLR lymphoid follicles were CD20+ B-cells, accounting for approximately 60% of the follicle. CD3+ T-cells accounted for almost 40% of the follicle (12). In addition to T and B-cells, networks of CD21+ follicular dendritic cells (FDC), which have important functions in the selection of memory B lymphocytes during GC reactions, are also found in CLR (26, 49). Within CLR, CD83+ mature DCs were most commonly found at the T-cell zone (50) whereas CD68+ cells, which have a role in clearing apoptotic B-cells, were scattered around the follicle (12). The co-localization of T-cells and CD83+ DCs within TLS has also been shown in other cancers (47). T follicular helper cells, which are characteristic of GCs, have not specifically been characterized within CLR but their density within the invasive margin has been shown to be related to CXCL13 expression in CRC (51). High endothelial venules (HEV) are characteristic of CLR and are more abundant next to tumor-associated lymphoid follicles than in normal mucosa-associated lymphoid follicles (12). HEV are thought to mediate recruitment of lymphocytes to the TME (26). Lymphatic vessels adjacent to CLR are also common and are hypothesized to facilitate efferent trafficking from CLR (12).

Taken together, the cellular composition and structure of CLR are similar to secondary lymphoid organs and suggest that CLR is a functional immunologic component contributing to the adaptive immune response to CRC. This is supported by the increased intratumoral lymphocytic infiltrate and gene expression signatures of cytotoxicity and DCs that are associated with CLR (38), as well as similar T-cell repertoires among CLR and TILs (52). Murine models have demonstrated a tumor-specific adaptive immune response (53, 54) that is driven by TLS even in the absence of secondary lymphoid structures (55). This host response can lead to tumor infiltration by immune cells and mediate tumor regression (54, 56, 57).

FOXP3+ regulatory T-cells (Tregs) comprise a small (<1%) percentage of CLR (12) but may play an important role in suppressing the anti-tumoral functions of CLR. Data from a murine lung adenocarcinoma model and human breast and prostate cancer suggests that Tregs may be preferentially recruited into TLS (37, 54, 58), which is supported by their preferential localization to the invasive margin of the tumor in CRC (51). Depletion of Tregs from TLS, via TGF-beta inhibition combined with an allogeneic tumor vaccine in a murine model of pancreatic cancer, promoted an anti-tumoral response (59).

Further insights into the cellular and spatial organization of CLR could be provided by highly multiplexed imaging mass cytometry, which can be used on fresh or formalin-fixed paraffin-embedded slides (60). Novel methods to sort and analyze the specific cellular components of TLS have been described in lung cancer (61) and could potentially be applied to CLR.

Induction and modification of TLS have been achieved in non-CRC human and murine studies, including in pancreatic cancer which is considered a non-immunogenic tumor (40, 53, 57, 59, 62). These exciting techniques, including tumor vaccines, Treg depletion, and cell line induced-TLS, have yet to be explored in CLR.

### Similarities and Differences Between CLR and CRC-Draining Lymph Nodes

CLR resembles secondary lymphoid structures, among which are tumor-draining lymph nodes (TDLN). TDLN are formed during embryogenesis and early life (63) whereas CLR forms as a response to CRC, lacks a capsule, and is found in the vicinity of the tumor, within the TME. Data comparing these two compartments of the host response in CRC is limited. Both TLS and TDLN can mediate neoantigen-specific responses (64–68). Data from a murine model of melanoma suggests that anti-tumor activity of TLS can be independent of TDLN or other secondary lymphoid organs (55).

TDLN have a paradoxical role in cancer immune surveillance and immune escape, in which cancer progression is characterized by a tumor-promoting milieu in TDLN (69), facilitating systemic tolerance to cancer antigens and predisposing to lymphatic metastasis. The reversal of this process is believed to contribute to the activity of certain ICI (70, 71). Later stages of CRC, including presence of lymphatic metastases, are also associated with decreased presence of CLR (7, 8), suggesting ineffective immune surveillance. The abundance of Tregs within TDLN is associated with lymphatic metastasis (72), akin to the evidence discussed above regarding the role of Tregs within CLR, and their depletion from both compartments augments the anti-tumor host response (59, 73, 74). CTLA-4 inhibition can induce anti-tumor immunity by intra-tumoral Treg reduction, without concurrent TDLN Treg reduction (75).

Frank “high jacking” of the immune milieu of CLR to promote cancer proliferation has not been demonstrated as it has in TDLN, but has been suggested in hepatocellular carcinoma (76). There is no data regarding the interaction of CLR with key factors that are thought to promote lymphatic metastasis, including members of the vascular endothelial growth factor (VEGF) secreted glycoproteins, as well as insulin and hepatocyte growth factors (77–79). Several cytokines, including CCL21 through the activation of CCR7 (80, 81), CCL5 and CXCL10 (82), have been implicated in metastatic lymphangiogenesis as well as the formation and maintenance of CLR and other tumor-specific immune responses.

### Metastatic Disease

Inspired by findings in primary CRC, studies have evaluated the lymphocytic infiltration of metastatic CRC. In liver metastases, T- and B- lymphocytic infiltration at the invasive margin have been associated with improved survival and response to chemotherapy (30, 31). CRC liver metastases may induce TLS with active GC within the metastatic margin, with evidence of B-cell priming and maturation at the metastatic site, including the detection of plasma cells (46).

In CRC lung metastases, a high density of TLS was demonstrated and the densities of CD8+ cytotoxic T-cells and DC were associated with improved survival (83). This contrasted with renal cell carcinoma (RCC), where TLS was scarce and the same immune infiltrates were associated with worse survival. These data demonstrate that quantification of TLS without structural and transcriptional characterization of the immune TME provides only partial insight into the immunological outcomes of TLS. An immunomodulatory role of TLS and a yet unknown interaction with metastases-infiltrating lymphocytes and other immune components may explain the contrasting survival associations with immune infiltrates among CRC and RCC.

The cellular composition of TLS associated with CRC lung metastases was assessed in a study with curative-intent pulmonary metastasectomy (84). Specifically, a high CD8+ density and high cytotoxic-to-regulatory T-cell ratio within metastases associated-TLS were associated with improved survival (84). In this study, only 7.1% of primary CRC was rated as having CLR, suggesting that the primary TME in patients with metastatic disease exhibits less immune activation. The paucity of CLR in patients with metastatic disease (84) in addition to evidence from prostate cancer raise the possibility that TLS regress with tumor progression (58), although others have demonstrated that B-lymphocytes increase with advancing tumor stage (51).

### Murine Models of CLR

Murine models that provide mechanistic insight regarding TLS have been described in a variety of conditions, including viral-induced ELS (36, 39, 43), and primary and metastatic cancers including pancreatic ductal adenocarcinoma (59), lung adenocarcinoma (54), hepatocellular carcinoma (76), melanoma (55, 85) and others. Murine models that specifically study CLR include the colitis-associated cancer model, created by exposure of mice to the carcinogen azoxymethane (AOM) and the mucosal irritant dextran sulfate (DSS) (49, 86). CLR in this model contained similar cell populations to human CLR and was enriched with lymphatic vessels and HEV, although GC morphology was not observed (26, 49). CXCL13 was highly expressed within CLR in this model and lymphocyte recruitment from the peripheral circulation to CLR was demonstrated using GFP-tagged murine splenocytes injected into the peripheral circulation (26).

Subcutaneous injections of the CRC cell line MC38 has also been used to study CLR (40, 57), although GCs were also not detected in this study (40). Unfortunately, neither of these models accurately reflects the pathogenesis and microenvironment of most human CRC. The absence of GCs in these models, which can be induced in murine viral associated lung TLS (36, 43), also raises the concern that they do not entirely recapitulate human CLR. An endoscopic orthotopic CRC model was developed by injecting the MC38 cell line into the colonic submucosa, creating tumors that are morphologically similar to human CRC (51). While this study did not focus on CLR *per se*, the authors were able to demonstrate CLR-like infiltrates in the invasive margin. In addition, endoscopic injection of CXCL13 resulted in tumor rejection in this model (51).

Caution is needed when interpreting data from non-CRC murine models. For example, the hepatocellular carcinoma (HCC) murine model showed that TLS promoted tumor formation and was associated with a worse prognosis (76). This suggests that the primary tumor and other components of the TME may alter the immunological function of TLS. The data discussed above regarding the difference between RCC and CRC lung metastases is another example of this difference (83). Research in CRC specific murine models is needed to better characterize the CLR and how CLR can be modified or manipulated to support and anti-tumor host response.

### Areas of Uncertainty and Future Directions

Despite the advances in our understanding of CLR and its utility as a prognostic marker, many questions remain. There is currently limited data about the utility of CLR as a predictive marker of response to immunotherapy or immunological adverse events. The interaction of CLR with pre-malignant colon lesions, the microbiome and the functional changes that occur within CLR during tumor progression are also currently unknown. Much remains to be elucidated regarding the interplay between CLR, TILs, the TME and TDLN. In depth mechanistic understanding will require murine models that can better recapitulate human CLR.

TCR sequencing, mass cytometry and single cell sequencing, are likely to advance our understanding of the immunological role that CLR plays in the TME. TCR sequencing of CLR can characterize the overlap between the T-cell repertoire of CLR and other immunological compartments, and support the efforts to uncover neoantigen-specific TCRs and improve adoptive T-cell therapy. Mass cytometry could expand on previous immunohistochemical characterization of CLR to better delineate cell populations and simultaneously visualize the structure, functional markers, and cellular interactions within CLR and the TME. Single-cell sequencing could provide insights into the heterogeneity of transcriptional programs as they relate to immune cell subtypes within CLR, and identify key drivers of immune surveillance and immune tolerance in the TME. Optimal application of these methods would be achieved in well-characterized clinical cohorts with informative molecular characterization, such as mutational burden, MSI status, consensus molecular subtype and intra-tumoral immune response.

Ultimately, the most important question will be if CLR can be leveraged to augment the immune response against cancer and improve patient outcomes. Attempts to systemically manipulate CLR will need to take potential tumor-promoting off-target effects into account, given the evidence of differing consequences of TLS formation across tissues, the role of TLS in autoimmunity (87), and the contribution of certain CLR-associated chemokines to lymphatic metastasis.

In conclusion, the Crohn's-like lymphoid reaction to colorectal cancer is a major component of the host immune response to cancer. CLR's diagnostic, predictive, and therapeutic implications need to be explored in greater detail.

## Author Contributions

AM and JG designed the manuscript, as well as acquired the images required for the figures. AM and MD wrote sections of the manuscript and created the figures. All authors contributed to manuscript revision, read, and approved the submitted version.

### Conflict of Interest Statement

The authors declare that the research was conducted in the absence of any commercial or financial relationships that could be construed as a potential conflict of interest.

## References

[1] Greenson JK Bonner JD Ben-Yzhak O Cohen HI Miselevich I Resnick MB. Phenotype of microsatellite unstable colorectal carcinomas: well-differentiated and focally mucinous tumors and the absence of dirty necrosis correlate with microsatellite instability. Am J Surg Pathol. (2003) 27:563–70. 10.1097/00000478-200305000-00001 12717242

[2] Greenson JK Huang SC Herron C Moreno V Bonner JD Tomsho LP. Pathologic predictors of microsatellite instability in colorectal cancer. Am J Surg Pathol. (2009) 33:126–33. 10.1097/PAS.0b013e31817ec2b1 18830122 PMC3500028

[3] Rozek LS Schmit SL Greenson JK Tomsho LP Rennert HS Rennert G Tumor-infiltrating lymphocytes, crohn's-like lymphoid reaction, and survival from colorectal cancer. J Natl Cancer Inst. (2016) 12:108 10.1093/jnci/djw027 PMC5017930 27172903

[4] Galon J Costes A Sanchez-Cabo F Kirilovsky A Mlecnik B Lagorce-Pages C. Type, density, and location of immune cells within human colorectal tumors predict clinical outcome. Science. (2006) 313:1960–4. 10.1126/science.1129139 17008531

[5] Mlecnik B Tosolini M Kirilovsky A Berger A Bindea G Meatchi T. Histopathologic-based prognostic factors of colorectal cancers are associated with the state of the local immune reaction. J Clin Oncol. (2011) 29:610–8. 10.1200/JCO.2010.30.5425 21245428

[6] Mlecnik B Bindea G Angell HK Maby P Angelova M Tougeron D. Integrative analyses of colorectal cancer show immunoscore is a stronger predictor of patient survival than microsatellite instability. Immunity. (2016) 44:698–711. 10.1016/j.immuni.2016.02.025 26982367

[7] Ogino S Nosho K Irahara N Meyerhardt JA Baba Y Shima K. Lymphocytic reaction to colorectal cancer is associated with longer survival, independent of lymph node count, microsatellite instability, and CpG island methylator phenotype. Clin Cancer Res. (2009) 15:6412–20. 10.1158/1078-0432.CCR-09-1438 19825961 PMC2771425

[8] Graham DM Appelman HD. Crohn's-like lymphoid reaction and colorectal carcinoma: a potential histologic prognosticator. Mod Pathol. (1990) 3:332–5. 2362940

[9] Dieu-Nosjean MC Goc J Giraldo NA Sautes-Fridman C Fridman WH. Tertiary lymphoid structures in cancer and beyond. Trends Immunol. (2014) 35:571–80. 10.1016/j.it.2014.09.006 25443495

[10] Harrison JC Dean PJ el-Zeky F Vander Zwaag R. Impact of the Crohn's-like lymphoid reaction on staging of right-sided colon cancer: results of multivariate analysis. Hum Pathol. (1995) 26:31–8. 10.1016/0046-8177(95)90111-6 7821914

[11] Kim JH Kim KJ Bae JM Rhee YY Cho NY Lee HS. Comparative validation of assessment criteria for Crohn-like lymphoid reaction in colorectal carcinoma. J Clin Pathol. (2015) 68:22–8. 10.1136/jclinpath-2014-202603 25322692

[12] Vayrynen JP Sajanti SA Klintrup K Makela J Herzig KH Karttunen TJ. Characteristics and significance of colorectal cancer associated lymphoid reaction. Int J Cancer. (2014) 134:2126–35. 10.1002/ijc.28533 24154855

[13] Ueno H Hashiguchi Y Shimazaki H Shinto E Kajiwara Y Nakanishi K. Objective criteria for crohn-like lymphoid reaction in colorectal cancer. Am J Clin Pathol. (2013) 139:434–41. 10.1309/AJCPWHUEFTGBWKE4 23525613

[14] Posch F Silina K Leibl S Mundlein A Moch H Siebenhuner A. Maturation of tertiary lymphoid structures and recurrence of stage II and III colorectal cancer. Oncoimmunology. (2018) 7:e1378844. 10.1080/2162402X.2017.1378844 29416939 PMC5798199

[15] Pages F Mlecnik B Marliot F Bindea G Ou FS Bifulco C. International validation of the consensus Immunoscore for the classification of colon cancer: a prognostic and accuracy study. Lancet. (2018) 391:2128–39. 10.1016/S0140-6736(18)30789-X 29754777

[16] Harrison JC Dean PJ el-Zeky F Vander Zwaag R. From Dukes through Jass: pathological prognostic indicators in rectal cancer. Hum Pathol. (1994) 25:498–505. 10.1016/0046-8177(94)90122-8 8200644

[17] Lewis B Lin J Wu X Xie H Shen B Lai K. Crohn's disease-like reaction predicts favorable prognosis in colitis-associated colorectal cancer. Inflamm Bowel Dis. (2013) 19:2190–8. 10.1097/MIB.0b013e31829e13e1 23917251

[18] Murphy J O'Sullivan GC Lee G Madden M Shanahan F Collins JK. The inflammatory response within Dukes' B colorectal cancers: implications for progression of micrometastases and patient survival. Am J Gastroenterol. (2000) 95:3607–14. 10.1111/j.1572-0241.2000.03377.x 11151900

[19] Hynes SO Coleman HG Kelly PJ Irwin S O'Neill RF Gray RT Back to the future: routine morphological assessment of the tumour microenvironment is prognostic in stage II/III colon cancer in a large population-based study. Histopathology. (2017) 71:12–26. 10.1111/his.13221 28165633

[20] Buckowitz A Knaebel HP Benner A Blaker H Gebert J Kienle P. Microsatellite instability in colorectal cancer is associated with local lymphocyte infiltration and low frequency of distant metastases. Br J Cancer. (2005) 92:1746–53. 10.1038/sj.bjc.6602534 15856045 PMC2362037

[21] Jenkins MA Hayashi S O'Shea AM Burgart LJ Smyrk TC Shimizu D. Pathology features in Bethesda guidelines predict colorectal cancer microsatellite instability: a population-based study. Gastroenterology. (2007) 133:48–56. 10.1053/j.gastro.2007.04.044 17631130 PMC2933045

[22] Overman MJ Lonardi S Wong KYM Lenz HJ Gelsomino F Aglietta M. Durable clinical benefit with nivolumab plus ipilimumab in DNA mismatch repair-deficient/microsatellite instability-High metastatic colorectal cancer. J Clin Oncol. (2018) 36:773–9. 10.1200/JCO.2017.76.9901 29355075

[23] Oyanagi H Shimada Y Nagahashi M Ichikawa H Tajima Y Abe K. SMAD4 alteration associates with invasive-front pathological markers and poor prognosis in colorectal cancer. Histopathology. (2019) 74:873–82. 10.1111/his.13805 30636020 PMC6849740

[24] Guinney J Dienstmann R Wang X de Reynies A Schlicker A Soneson C. The consensus molecular subtypes of colorectal cancer. Nat Med. (2015) 21:1350–6. 26457759 10.1038/nm.3967 PMC4636487

[25] Becht E de Reynies A Giraldo NA Pilati C Buttard B Lacroix L. Immune and stromal classification of colorectal cancer is associated with molecular subtypes and relevant for precision immunotherapy. Clin Cancer Res. (2016) 22:4057–66. 10.1158/1078-0432.CCR-15-2879 26994146

[26] Di Caro G Bergomas F Grizzi F Doni A Bianchi P Malesci A. Occurrence of tertiary lymphoid tissue is associated with T-cell infiltration and predicts better prognosis in early-stage colorectal cancers. Clin Cancer Res. (2014) 20:2147–58. 10.1158/1078-0432.CCR-13-2590 24523438

[27] Sjoquist KM Renfro LA Simes RJ Tebbutt NC Clarke S Seymour MT. Personalizing survival predictions in advanced colorectal cancer: the ARCAD nomogram project. J Natl Cancer Inst. (2018) 110:638–48. 10.1093/jnci/djx253 29267900 PMC6005015

[28] Masugi Y Nishihara R Hamada T Song M da Silva A Kosumi K. Tumor PDCD1LG2 (PD-L2) expression and the lymphocytic reaction to colorectal cancer. Cancer Immunol Res. (2017) 5:1046–55. 10.1158/2326-6066.CIR-17-0122 29038297 PMC5668177

[29] Weinstein AM Giraldo NA Petitprez F Julie C Lacroix L Peschaud F. Association of IL-36gamma with tertiary lymphoid structures and inflammatory immune infiltrates in human colorectal cancer. Cancer Immunol Immunother. (2019) 68:109–20. 10.1007/s00262-018-2259-0 30315348 PMC7185158

[30] Halama N Michel S Kloor M Zoernig I Pommerencke T von Knebel Doeberitz M. The localization and density of immune cells in primary tumors of human metastatic colorectal cancer shows an association with response to chemotherapy. Cancer Immun. (2009) 9:1. 19226101 PMC2935770

[31] Halama N Michel S Kloor M Zoernig I Benner A Spille A. Localization and density of immune cells in the invasive margin of human colorectal cancer liver metastases are prognostic for response to chemotherapy. Cancer Res. (2011) 71:5670–7. 10.1158/0008-5472.CAN-11-0268 21846824

[32] Alexander J Watanabe T Wu TT Rashid A Li S Hamilton SR. Histopathological identification of colon cancer with microsatellite instability. Am J Pathol. (2001) 158:527–35. 10.1016/S0002-9440(10)63994-6 11159189 PMC1850324

[33] Suzuki A Masuda A Nagata H Kameoka S Kikawada Y Yamakawa M. Mature dendritic cells make clusters with T cells in the invasive margin of colorectal carcinoma. J Pathol. (2002) 196:37–43. 10.1002/path.1018 11748640

[34] Gianotti L Sargenti M Galbiati F Nespoli L Brivio F Rescigno M. Phenotype and function of dendritic cells and T-lymphocyte polarization in the human colonic mucosa and adenocarcinoma. Eur J Surg Oncol. (2008) 34:883–9. 10.1016/j.ejso.2008.01.026 18325725

[35] Yuan A Steigen SE Goll R Vonen B Husbekk A Cui G. Dendritic cell infiltration pattern along the colorectal adenoma-carcinoma sequence. Apmis. (2008) 116:445–56. 10.1111/j.1600-0463.2008.00879.x 18754318

[36] Halle S Dujardin HC Bakocevic N Fleige H Danzer H Willenzon S. Induced bronchus-associated lymphoid tissue serves as a general priming site for T cells and is maintained by dendritic cells. J Exp Med. (2009) 206:2593–601. 10.1084/jem.20091472 19917776 PMC2806625

[37] Gobert M Treilleux I Bendriss-Vermare N Bachelot T Goddard-Leon S Arfi V. Regulatory T cells recruited through CCL22/CCR4 are selectively activated in lymphoid infiltrates surrounding primary breast tumors and lead to an adverse clinical outcome. Cancer Res. (2009) 69:2000–9. 10.1158/0008-5472.CAN-08-2360 19244125

[38] Coppola D Nebozhyn M Khalil F Dai H Yeatman T Loboda A. Unique ectopic lymph node-like structures present in human primary colorectal carcinoma are identified by immune gene array profiling. Am J Pathol. (2011) 179:37–45. 10.1016/j.ajpath.2011.03.007 21703392 PMC3123872

[39] GeurtsvanKessel CH Willart MA Bergen IM van Rijt LS Muskens F Elewaut D. Dendritic cells are crucial for maintenance of tertiary lymphoid structures in the lung of influenza virus-infected mice. J Exp Med. (2009) 206:2339–49. 10.1084/jem.20090410 19808255 PMC2768850

[40] Weinstein AM Chen L Brzana EA Patil PR Taylor JL Fabian KL. Tbet and IL-36gamma cooperate in therapeutic DC-mediated promotion of ectopic lymphoid organogenesis in the tumor microenvironment. Oncoimmunology. (2017) 6:e1322238. 10.1080/2162402X.2017.1322238 28680760 PMC5486180

[41] Bar-Ephraim YE Mebius RE. Innate lymphoid cells in secondary lymphoid organs. Immunol Rev. (2016) 271:185–99. 10.1111/imr.12407 27088915

[42] Jones GW Hill DG Jones SA. Understanding immune cells in tertiary lymphoid organ development: it is all starting to come together. Front Immunol. (2016) 7:401. 10.3389/fimmu.2016.00401 27752256 PMC5046062

[43] Denton AE Innocentin S Carr EJ Bradford BM Lafouresse F Mabbott NA. Type I interferon induces CXCL13 to support ectopic germinal center formation. J Exp Med. (2019) 216:621–37. 10.1084/jem.20181216 30723095 PMC6400543

[44] Silina K Soltermann A Attar FM Casanova R Uckeley ZM Thut H. Germinal centers determine the prognostic relevance of tertiary lymphoid structures and are impaired by corticosteroids in lung squamous cell carcinoma. Cancer Res. (2018) 78:1308–20. 10.1158/0008-5472.CAN-17-1987 29279354

[45] Germain C Gnjatic S Dieu-Nosjean MC. Tertiary lymphoid structure-associated B cells are key players in Anti-tumor immunity. Front Immunol. (2015) 6:67. 10.3389/fimmu.2015.00067 25755654 PMC4337382

[46] Meshcheryakova A Tamandl D Bajna E Stift J Mittlboeck M Svoboda M. B cells and ectopic follicular structures: novel players in anti-tumor programming with prognostic power for patients with metastatic colorectal cancer. PLoS ONE. (2014) 9:e99008. 10.1371/journal.pone.0099008 24905750 PMC4048213

[47] Dieu-Nosjean MC Giraldo NA Kaplon H Germain C Fridman WH Sautes-Fridman C. Tertiary lymphoid structures, drivers of the anti-tumor responses in human cancers. Immunol Rev. (2016) 271:260–75. 10.1111/imr.12405 27088920

[48] Klintrup K Makinen JM Kauppila S Vare PO Melkko J Tuominen H. Inflammation and prognosis in colorectal cancer. Eur J Cancer. (2005) 41:2645–54. 10.1016/j.ejca.2005.07.017 16239109

[49] Bergomas F Grizzi F Doni A Pesce S Laghi L Allavena P. Tertiary intratumor lymphoid tissue in colo-rectal cancer. Cancers. (2011) 4:1–10. 10.3390/cancers4010001 24213222 PMC3712686

[50] McMullen TP Lai R Dabbagh L Wallace TM de Gara CJ. Survival in rectal cancer is predicted by T cell infiltration of tumour-associated lymphoid nodules. Clin Exp Immunol. (2010) 161:81–8. 10.1111/j.1365-2249.2010.04147.x 20408858 PMC2940152

[51] Bindea G Mlecnik B Tosolini M Kirilovsky A Waldner M Obenauf AC. Spatiotemporal dynamics of intratumoral immune cells reveal the immune landscape in human cancer. Immunity. (2013) 39:782–95. 10.1016/j.immuni.2013.10.003 24138885

[52] Maoz A Greenson JK Melas M Emerson RO Vignali M Robins H Similar T-cell repertoires of tumor infiltrating lymphocytes and Crohn's-like lymphoid reaction in colorectal cancer. J Clin Oncol. (2017) 35(15_suppl):e15133–e. 10.1200/JCO.2017.35.15_suppl.e15133

[53] Shimizu K Yamasaki S Shinga J Sato Y Watanabe T Ohara O. Systemic DC activation modulates the tumor microenvironment and shapes the long-lived tumor-specific memory mediated by CD8+ T cells. Cancer Res. (2016) 76:3756–66. 10.1158/0008-5472.CAN-15-3219 27371739

[54] Joshi NS Akama-Garren EH Lu Y Lee DY Chang GP Li A. Regulatory T cells in tumor-associated tertiary lymphoid structures suppress anti-tumor T cell responses. Immunity. (2015) 43:579–90. 10.1016/j.immuni.2015.08.006 26341400 PMC4826619

[55] Schrama D Voigt H Eggert AO Xiang R Zhou H Schumacher TN. Immunological tumor destruction in a murine melanoma model by targeted LTalpha independent of secondary lymphoid tissue. Cancer Immunol Immunother. (2008) 57:85–95. 10.1007/s00262-007-0352-x 17605009 PMC11030041

[56] Hindley JP Jones E Smart K Bridgeman H Lauder SN Ondondo B. T-cell trafficking facilitated by high endothelial venules is required for tumor control after regulatory T-cell depletion. Cancer Res. (2012) 72:5473–82. 10.1158/0008-5472.CAN-12-1912 22962270 PMC3491872

[57] Zhu G Nemoto S Mailloux AW Perez-Villarroel P Nakagawa R Falahat R. Induction of tertiary lymphoid structures with antitumor function by a lymph node-derived stromal cell line. Front Immunol. (2018) 9:1609. 10.3389/fimmu.2018.01609 30061886 PMC6054958

[58] Garcia-Hernandez ML Uribe-Uribe NO Espinosa-Gonzalez R Kast WM Khader SA Rangel-Moreno J. A Unique cellular and molecular microenvironment is present in tertiary lymphoid organs of patients with spontaneous prostate cancer regression. Front Immunol. (2017) 8:563. 10.3389/fimmu.2017.00563 28567040 PMC5434117

[59] Soares KC Rucki AA Kim V Foley K Solt S Wolfgang CL. TGF-beta blockade depletes T regulatory cells from metastatic pancreatic tumors in a vaccine dependent manner. Oncotarget. (2015) 6:43005–15. 10.18632/oncotarget.5656 26515728 PMC4767487

[60] Singh M Chaudhry P Gerdtsson E Maoz A Cozen W Hicks J Highly multiplexed imaging mass cytometry allows visualization of tumor and immune cell interactions of the tumor microenvironment in FFPE tissue sections. Blood. (2017) 130(Suppl. 1):2751.

[61] Devi-Marulkar P Kaplon H Dieu-Nosjean MC Lawand M. Designed methods for the sorting of tertiary lymphoid structure-immune cell populations. Methods Mol Biol. (2018) 1845:189–204. 10.1007/978-1-4939-8709-2_11 30141014

[62] Lutz ER Wu AA Bigelow E Sharma R Mo G Soares K. Immunotherapy converts nonimmunogenic pancreatic tumors into immunogenic foci of immune regulation. Cancer Immunol Res. (2014) 2:616–31. 10.1158/2326-6066.CIR-14-0027 24942756 PMC4082460

[63] Hiraoka N Ino Y Yamazaki-Itoh R. Tertiary lymphoid organs in cancer tissues. Front Immunol. (2016) 7:244. 10.3389/fimmu.2016.00244 27446075 PMC4916185

[64] Dunn GP Bruce AT Ikeda H Old LJ Schreiber RD. Cancer immunoediting: from immunosurveillance to tumor escape. Nat Immunol. (2002) 3:991–8. 10.1038/ni1102-991 12407406

[65] Wang H Nemoto-Sasaki Y Kondo T Akiyama M Mukaida N. Potential involvement of monocyte chemoattractant protein (MCP)-1/CCL2 in IL-4-mediated tumor immunity through inducing dendritic cell migration into the draining lymph nodes. Int Immunopharmacol. (2003) 3:627–42. 10.1016/S1567-5769(02)00251-5 12757733

[66] Vermi W Micheletti A Lonardi S Costantini C Calzetti F Nascimbeni R. slanDCs selectively accumulate in carcinoma-draining lymph nodes and marginate metastatic cells. Nat Commun. (2014) 5:3029. 10.1038/ncomms4029 24398631

[67] Allen F Rauhe P Askew D Tong AA Nthale J Eid S. CCL3 Enhances antitumor immune priming in the lymph node via IFNgamma with dependency on natural killer cells. Front Immunol. (2017) 8:1390. 10.3389/fimmu.2017.01390 29109732 PMC5660298

[68] Yoshizawa H Sakai K Chang AE Shu SY. Activation by anti-CD3 of tumor-draining lymph node cells for specific adoptive immunotherapy. Cell Immunol. (1991) 134:473–9. 10.1016/0008-8749(91)90318-6 1827049

[69] Munn DH Mellor AL. The tumor-draining lymph node as an immune-privileged site. Immunol Rev. (2006) 213:146–58. 10.1111/j.1600-065X.2006.00444.x 16972902

[70] Fransen MF Schoonderwoerd M Knopf P Camps MG Hawinkels LJ Kneilling M. Tumor-draining lymph nodes are pivotal in PD-1/PD-L1 checkpoint therapy. JCI Insight. (2018) 3:e124507. 10.1172/jci.insight.124507 30518694 PMC6328025

[71] Hebb JPO Mosley AR Vences-Catalan F Rajasekaran N Rosen A Ellmark P. Administration of low-dose combination anti-CTLA4, anti-CD137, and anti-OX40 into murine tumor or proximal to the tumor draining lymph node induces systemic tumor regression. Cancer Immunol Immunother. (2018) 67:47–60. 10.1007/s00262-017-2059-y 28905118 PMC7446289

[72] Deng L Zhang H Luan Y Zhang J Xing Q Dong S. Accumulation of foxp3+ T regulatory cells in draining lymph nodes correlates with disease progression and immune suppression in colorectal cancer patients. Clin Cancer Res. (2010) 16:4105–12. 10.1158/1078-0432.CCR-10-1073 20682706

[73] Boissonnas A Scholer-Dahirel A Simon-Blancal V Pace L Valet F Kissenpfennig A. Foxp3+ T cells induce perforin-dependent dendritic cell death in tumor-draining lymph nodes. Immunity. (2010) 32:266–78. 10.1016/j.immuni.2009.11.015 20137985

[74] Keenan BP Saenger Y Kafrouni MI Leubner A Lauer P Maitra A. A Listeria vaccine and depletion of T-regulatory cells activate immunity against early stage pancreatic intraepithelial neoplasms and prolong survival of mice. Gastroenterology. (2014) 146:1784–94.e6. 10.1053/j.gastro.2014.02.055 24607504 PMC4035450

[75] Selby MJ Engelhardt JJ Quigley M Henning KA Chen T Srinivasan M. Anti-CTLA-4 antibodies of IgG2a isotype enhance antitumor activity through reduction of intratumoral regulatory T cells. Cancer Immunol Res. (2013) 1:32–42. 10.1158/2326-6066.CIR-13-0013 24777248

[76] Finkin S Yuan D Stein I Taniguchi K Weber A Unger K. Ectopic lymphoid structures function as microniches for tumor progenitor cells in hepatocellular carcinoma. Nat Immunol. (2015) 16:1235–44. 10.1038/ni.3290 26502405 PMC4653079

[77] Langheinrich MC Schellerer V Perrakis A Lohmuller C Schildberg C Naschberger E. Molecular mechanisms of lymphatic metastasis in solid tumors of the gastrointestinal tract. Int J Clin Exp Pathol. (2012) 5:614–23. 10.5772/30355 22977656 PMC3438765

[78] Achen MG Stacker SA. Molecular control of lymphatic metastasis. Ann N Y Acad Sci. (2008) 1131:225–34. 10.1196/annals.1413.020 18519975

[79] Kawada K Hosogi H Sonoshita M Sakashita H Manabe T Shimahara Y. Chemokine receptor CXCR3 promotes colon cancer metastasis to lymph nodes. Oncogene. (2007) 26:4679–88. 10.1038/sj.onc.1210267 17297455

[80] Hu X Luo J. Heterogeneity of tumor lymphangiogenesis: progress and prospects. Cancer Sci. (2018) 109:3005–12. 10.1111/cas.13738 30007095 PMC6172057

[81] Li S Li Q. Cancer stem cells, lymphangiogenesis, and lymphatic metastasis. Cancer Lett. (2015) 357:438–47. 10.1016/j.canlet.2014.12.013 25497008

[82] Farnsworth RH Karnezis T Maciburko SJ Mueller SN Stacker SA. The Interplay Between Lymphatic Vessels and Chemokines. Front Immunol. (2019) 10:518. 10.3389/fimmu.2019.00518 31105685 PMC6499173

[83] Remark R Alifano M Cremer I Lupo A Dieu-Nosjean MC Riquet M. Characteristics and clinical impacts of the immune environments in colorectal and renal cell carcinoma lung metastases: influence of tumor origin. Clin Cancer Res. (2013) 19:4079–91. 10.1158/1078-0432.CCR-12-3847 23785047

[84] Schweiger T Berghoff AS Glogner C Glueck O Rajky O Traxler D. Tumor-infiltrating lymphocyte subsets and tertiary lymphoid structures in pulmonary metastases from colorectal cancer. Clin Exp Metastasis. (2016) 33:727–39. 10.1007/s10585-016-9813-y 27449756 PMC5035322

[85] Rodriguez AB Peske JD Engelhard VH. Identification and Characterization of Tertiary Lymphoid Structures in Murine Melanoma. Methods Mol Biol. (2018) 1845:241–57. 10.1007/978-1-4939-8709-2_14 30141017 PMC6269110

[86] Snider AJ Bialkowska AB Ghaleb AM Yang VW Obeid LM Hannun YA. Murine Model for Colitis-Associated Cancer of the Colon. Methods Mol Biol. (2016) 1438:245–54. 10.1007/978-1-4939-3661-8_14 27150094 PMC5657253

[87] Weinstein JS Delano MJ Xu Y Kelly-Scumpia KM Nacionales DC Li Y. Maintenance of anti-Sm/RNP autoantibody production by plasma cells residing in ectopic lymphoid tissue and bone marrow memory B cells. J Immunol. (2013) 190:3916–27. 10.4049/jimmunol.1201880 23509349 PMC3622197

